# Identification and validation of a novel glycolysis-related ceRNA network for sepsis-induced cardiomyopathy

**DOI:** 10.3389/fmed.2024.1343281

**Published:** 2024-02-19

**Authors:** Lulu Cheng, Jiabin Liang, Fangmei Xie, Zeping Han, Wenfeng Luo, Hanwei Chen, Jinhua He

**Affiliations:** ^1^Postgraduate Cultivation Base of Guangzhou University of Chinese Medicine, Panyu Central Hospital, Guangzhou, China; ^2^Central Laboratory, Guangzhou Panyu Central Hospital, Guangzhou, China; ^3^Radiology Department of Panyu Health Management Center (Panyu Rehabilitation Hospital), Guangzhou, China

**Keywords:** sepsis-induced cardiomyopathy, glycolysis, ceRNA network, bioinformatics analysis, IER3

## Abstract

**Purpose:**

Sepsis-induced cardiomyopathy (SIC) is a major life-threatening condition in critically infected patients. Early diagnosis and intervention are important to improve patient prognosis. Recognizing the pivotal involvement of the glycolytic pathway in SIC, this study aims to establish a glycolysis-related ceRNA network and explore novel diagnostic avenues.

**Materials and methods:**

SIC-related datasets were carefully filtered from the GEO database. CytoHubba was used to identify differentially expressed genes (DEGs) associated with glycolysis. A predictive method was then used to construct an lncRNA-miRNA-mRNA network. Dual-luciferase reporter assays validated gene interactions, and the specificity of this ceRNA network was confirmed in peripheral blood mononuclear cells (PBMCs) from SIC patients. Logistic analysis was used to examine the correlation between the ceRNA network and SIC. Diagnostic potential was assessed using receiver operating characteristic (ROC) curves, and correlation analysis investigated any associations between gene expression and clinical indicators.

**Results:**

IER3 was identified as glycolysis-related DEG in SIC, and a ceRNA network (SNHG17/miR-214-3p/IER3) was established by prediction. Dual luciferase reporter gene assay confirmed the presence of mutual binding between IER3, miR-214-3p and SNHG17. RT-qPCR verified the specific expression of this ceRNA network in SIC patients. Multivariate logistic analysis established the correlation between the ceRNA network and SIC. ROC analysis demonstrated its high diagnostic specificity (AUC > 0.8). Correlation analysis revealed a negative association between IER3 expression and oxygenation index in SIC patients (*p* < 0.05). Furthermore, miR-214-3p expression showed a negative correlation with NT-proBNP (*p* < 0.05).

**Conclusion:**

In this study, we identified and validated a ceRNA network associated with glycolysis in SIC: SNHG17/miR-214-3p/IER3. This ceRNA network may play a critical role in the onset and development of SIC. This finding is important to further our understanding of the pathophysiological mechanisms underlying SIC and to explore potential diagnostic and therapeutic targets for SIC.

## 1 Introduction

Sepsis is a common and complex medical condition, currently defined as an unbalanced response by the body to the invasion of harmful microorganisms, like bacteria, resulting in systemic inflammation and acute organ dysfunction (1). Among the myriad clinical consequences of sepsis, sepsis-induced cardiomyopathy (SIC) stands out prominently. It is characterized by ventricular dilatation, poor contractility, and decreased ejection fraction (2, 3). Unfortunately, this cardiac complication significantly contributes to the already high mortality rate associated with sepsis, leading to a considerably worse prognosis. Septic patients without SIC face a mortality rate of approximately 20%, while those with SIC experience a strikingly higher mortality rate ranging from 70 to 90% (4).

Recognizing the critical implications of SIC, early diagnosis becomes paramount for effective patient management. However, the existing clinical diagnostic methods for SIC, primarily relying on conventional indicators like transthoracic echocardiography, atrial natriuretic peptide, and cardiac troponin (5), lack the specificity required for prompt identification and treatment of SIC (6). Therefore, elucidating the molecular mechanisms underlying the occurrence and development of SIC is of utmost importance, offering promising targets for its prevention, diagnosis, and treatment.

The development of SIC is intricately associated with aberrations in myocardial cell metabolism. Glucose serves as the primary metabolic substrate for myocardial cells, primarily undergoing glycolysis and oxidative phosphorylation (OXPHOS) to produce ATP, thereby providing energy support for myocardial cells (7). In the normal heart, the majority of ATP is derived from mitochondrial OXPHOS, whereas glycolysis and lactate oxidation account for only 10–40% of ATP production (8, 9). However, in the context of sepsis, there is an imbalance in myocardial energy metabolism (10). Myocardial mitochondrial fatty acid oxidation is disrupted, leading to an accelerated rate of aerobic glycolysis, excessive glucose consumption, and accumulation of pyruvate in the myocardium (11). This worsens the heart’s function in sepsis (12). In a word, glycolysis plays a significant role and may offer new avenues for early diagnosis and treatment of SIC.

The theory of Competing Endogenous RNAs (ceRNA) was initially proposed by Salmena et al. (13). This theory suggests that different RNA molecules can competitively bind to microRNAs (miRNA), thereby disrupting their inhibition of target genes and regulating gene expression (13). Long non-coding RNAs (lncRNAs) have been shown to act as ceRNAs by competitively sequestering miRNAs to modulate the expression of target genes (14). They play regulatory roles in various diseases, including sepsis (15) and SIC (16). Using bioinformatics techniques, we can identify hub DEGs in SIC and build specific ceRNA regulatory networks using high-throughput sequencing tools. This approach contributes to a deeper understanding of the pathogenic mechanisms of SIC and aids in the discovery of new methods for early prediction and diagnosis of SIC.

**Figure 1:**
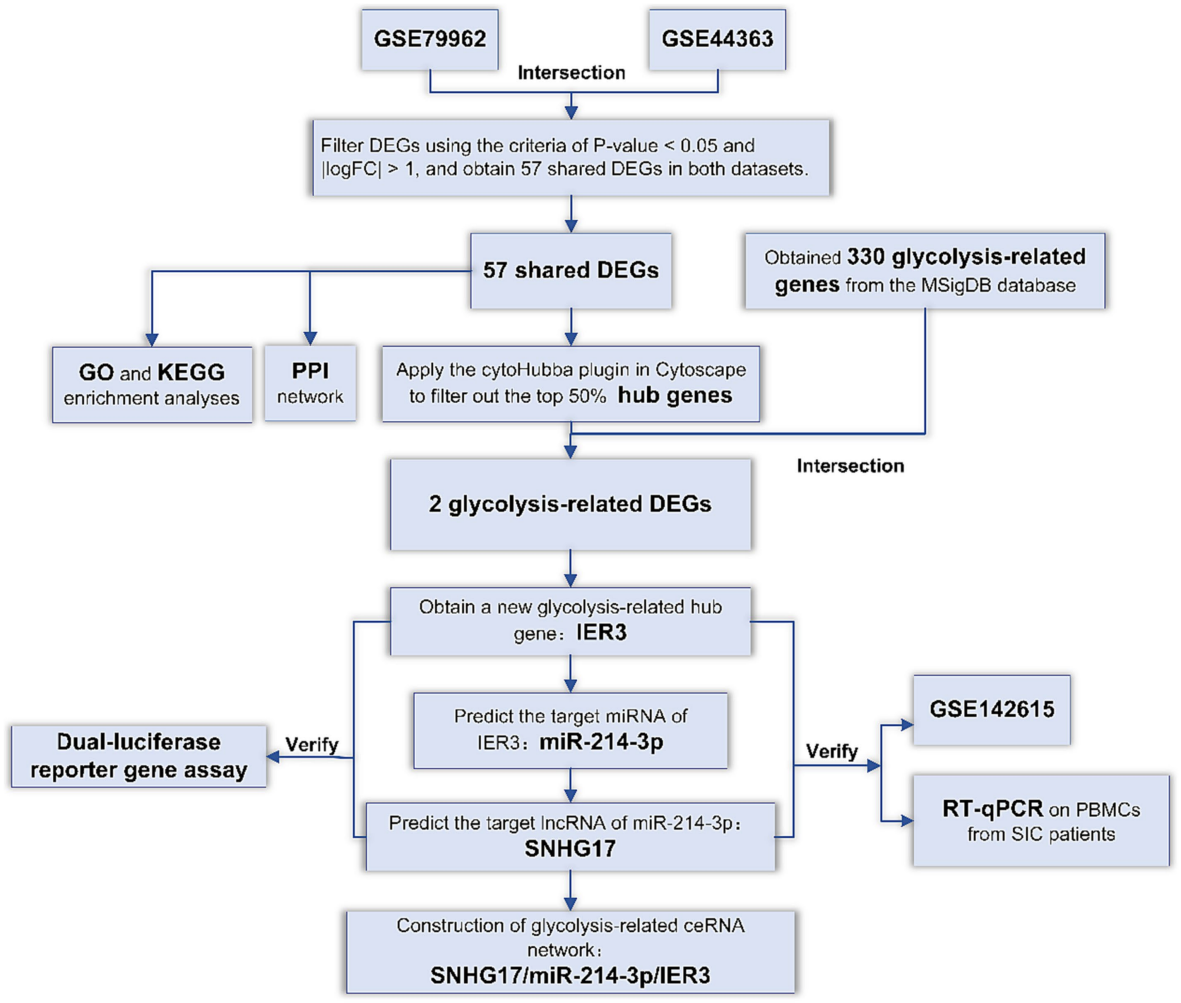
Flowchart of the study.

DEGs: Differentially expressed genes; GO: Gene Ontology; KEGG: Kyoto Encyclopedia of Genes and Genomes; PPI: protein–protein interaction; RT-qPCR: Reverse transcription-quantitative real-time PCR.

In order to explore specific genes related to glycolysis in SIC, we first screened for common DEGs in SIC datasets from the GEO database. Subsequently, we identified pivotal genes associated with glycolysis among these DEGs. Utilizing multiple databases, we predicted the miRNAs and lncRNAs targeted and constructed a ceRNA network related to glycolysis. To validate specific binding within the ceRNA network, dual-luciferase reporter gene assays were performed. In addition, we verified the specific expression of ceRNAs in SIC patients by RT-qPCR experiments using peripheral blood mononuclear cell (PBMC) samples. The accuracy of these ceRNAs in diagnosing SIC was evaluated using ROC curves. The flowchart representing the methodology of this study is shown in Figure 1.

## 2 Materials and methods

### 2.1 Bioinformatics analysis

#### 2.1.1 Selection of microarray dataset

We selected 5 microarray datasets related to SIC from the GEO database (17),^1^ as shown in Table 1. Among these, the GSE79962 series (including 20 human SIC samples and 11 non-SIC samples) and GSE44363 (including 4 mouse SIC samples and 4 non-SIC samples) were used as the training set. The GSE142615 series (comprising 4 mouse SIC samples and 4 non-SIC samples) contains both mRNA and lncRNA data and was used as the validation set.

**Table 1:** Information on the datasets utilized in this study.

GEO number	Platform	Species	Source tissue	Sample (SIC/control)	Data	Attribute
GSE79962	GPL6244	Human	Heart	20/11	mRNA	Test set
GSE44363	GPL1261	Mice	Heart	4/4	mRNA	Test set
GSE142615	GPL27951	Mice	Heart	4/4	mRNA and lncRNA	Validation set

#### 2.1.2 Extraction of DEGs

To identify and analyze the DEGs between the SIC and control groups within the GEO datasets, we utilized the GEO2R online tool.^2^ GEO2R is an online tool supplied by the GEO database that analyzes and visualizes GEO data using R programming, presenting results in a gene table sorted by importance (17). We set the criteria for DEGs selection as follows: *p* < 0.05 and |logFC| > 1 to identify DEGs with significant expression differences. Subsequently, we used the online Venn tool (18)^3^ to identify DEGs that were common to both datasets.

#### 2.1.3 Functional enrichment analysis of DEGs and PPI analysis

We assessed the biological functions of the identified DEGs using the DAVID Bioinformatics Resources (19).^4^ We generated the PPI network using the STRING database (20)^5^ with a minimum required interaction score set to 0.4. The obtained PPI information was then imported into Cytoscape 3.7.1 software (21).^6^ We used the cytoHubba plugin within Cytoscape to identify important DEGs as hub genes in the PPI network. The cytoHubba plugin employs various topological algorithms to predict and explore key nodes and subnetworks within a given network (22). Therefore, we applied the MCC algorithm and selected the top 50% ranked genes as hub genes for further analysis.

#### 2.1.4 Selection of glycolysis-related hub genes

We first identified five gene sets related to glycolysis from MSigDB^7^ (23): BIOCARTA_GLYCOLYSIS_PATHWAY,GO_GLYCOLYTIC_PROCESS, HALLMARK_GLYCOLYSIS, KEGG_GLYCOLYSIS_GLUCONEOGENESIS, and REACTOME_GLYCOLYSIS. After merging and removing duplicates from these five gene sets, we obtained a total of 330 glycolysis-related genes (Supplementary Table 1: S1). We then used Venn diagrams to identify the intersection between the glycolysis-related gene sets and the hub genes, leading to the selection of glycolysis-related hub genes. Finally, we conducted validation of the selected GRHGs using the dataset GSE142615.

#### 2.1.5 Construction of the ceRNA network

We predicted miRNAs related to GRHGs using three online miRNA databases: TarBase (24),^8^ starBase (25),^9^ and miRWalk (26).^10^ The intersection of miRNA predictions from these three databases was obtained using the Venn tool. Subsequently, we used RNA22 (27)^11^ to predict lncRNAs that interact with the target miRNAs. We validated the predictions using dataset GSE142615 and selected a subset of lncRNAs with |logFC| > 1 from the validation results.

### 2.2 The dual-luciferase reporter gene assay

Through the TargetScan website^12^ (28), the binding sites of IER3/miR-214-3p and miR-214-3p/SNHG17 were predicted. Wild-type and mutant PCR primers were designed accordingly. HEK293-T cells DNA was extracted as the template, and PCR was performed to amplify the 3’UTR sequences of IER3 and SNHG17. After amplification, the PCR products were subjected to enzymatic digestion, and the digested products were purified following the instructions of the Gel Extraction Kit (DONGSHENG BIOTECH, China). The purified digested products underwent ligation reactions and were separately introduced into *Escherichia coli* DH5α competent cells. Single colonies were picked for amplification, and plasmid extraction was carried out using the Plasmid Isolation Kit (DONGSHENG BIOTECH, China) following the instructions. The resulting plasmids were identified by enzymatic digestion and separated on a 1% agarose gel with ethidium bromide. Positive clones were confirmed and subsequent sequencing of the plasmids was performed.

HEK293-T cells are widely used as a functional cell for producing adenovirus vectors, adeno-associated virus vectors and cellular biology research. The day before transfection, HEK293-T cells were seeded at a density of 2 × 10^4^ cells per well in a 24-well plate using DMEM high-glucose medium (Gibico, United States) containing 10% FBS. On the day of transfection, when the cell confluence reached approximately 50–60%, each well was treated with 1 μL of cellfectin II Reagent (Invitrogen) diluted in OPTI-MEM medium (Gibico, United States). Subsequently, a mixture containing 20 μM miR-214-3p mimic or miR-214-3p inhibitor (Guangzhou RiboBio, China) and 0.5 μg of wild-type or mutant plasmid was added to each well. Negative control (NC) groups with empty plasmid and NC inhibitor were also set up with three replicates for each group. The medium was changed for new growth medium after 6 h of transfection, and the cells were then cultured for a further 48 h. After that, the cells were extracted, and a GloMax bioluminescence detector was used to quantify the activity of firefly luciferase and Renilla luciferase. The measurements were performed following the instructions provided in the Promega Dual-Luciferase System Kit (Promega, United States), and the values for firefly luciferase and Renilla luciferase activities were recorded.

### 2.3 Clinical sample collection and RT-qPCR

PBMC samples from SIC patients were collected from the Panyu Central Hospital in Guangzhou, China. This study strictly adhered to the Helsinki Declaration and relevant legal and regulatory requirements, and it was approved by the Ethics Committee of Panyu Central Hospital (Approval No: PYRC-2023-086). Informed consent was obtained from all participants before the study commenced. Inclusion criteria were as follows: (1) Patients diagnosed with sepsis according to the Sepsis-3 criteria upon admission (1); (2) Patients with sepsis exhibiting the following conditions: left ventricular ejection fraction (LVEF) ≤ 50% and elevated myocardial injury markers; (3) Inclusion within a time frame of no more than 7 days from the initial diagnosis of SIC. Exclusion criteria were as follows: (1) Age < 18 years; (2) Concomitant acute myocardial infarction or severe arrhythmias; (3) History of chronic heart failure or chronic renal insufficiency; (4) End-stage tumor or hematological malignancies.

Each participant collected a 5 mL venous blood sample, which was stored at 4°C for a short period of time. PBMC was extracted on the day of blood collection and RNA was extracted using the TRIzol method. Subsequently, RNA was reverse transcribed into cDNA. RNA samples for IER3 and SNHG17 determination were reverse transcribed using the Hifair^®^ first-strand cDNA synthesis kit (Yeasen, China), while miR-214-3p RNA samples were processed using the miRNA 1st strand cDNA synthesis kit (Accurate Biotechnology, China). The miR-214-3p stem-loop primer sequence was: GTCGTATCCAGTGCAGGGTCCGAGGTATTCGCACTGGATACGACACTGCC. After reverse transcription, qPCR experiments were performed using ChamQ Universal SYBR qPCR Master Mix (Vazyme, China). GAPDH was used as the internal reference gene for IER3 and SNHG17, and U6 served as the internal reference gene for miR-214-3p. The relative expression of target genes was analyzed using the 2-ΔΔCt method. Primer sequences are shown in Table 2, and all experiments were performed in triplicate.

**Table 2:** Primer sequence.

Genes	Forward primer (5′ to 3′)	Reverse primer (5′ to 3′)
IER3	GCAGCCGCAGGGTTCTCTACC	CTCTTCAGCCATCAGGATCTGG
miR-214-3p	GGCACAGCAGGCACAGACA	AGTGCAGGGTCCGAGGTATT
SNHG17	TGGGATCTGGGTTTGCTGATATTTCT	GGTAGCCTCACTCTCCATTCTCTG
U6	CTCGCTTCGGCAGCACA	AACGCTTCACGAATTTGCGT
GAPDH	AGAAGGCTGGGGCTCATTTG	GCAGGAGGCATTGCTGATGAT

### 2.4 Statistical analyses

Statistical analyses were conducted using SPSS 25 and GraphPad Prism 9.5.1 software, and all data are presented as mean ± standard deviation. When comparing data between two groups, paired *t*-tests were used for data that followed a normal distribution, and non-parametric tests were used for data that did not follow a normal distribution. ANOVA was used for comparisons of relative luciferase activity among different groups. Univariate and multifactorial logistic regression analyses were performed to determine the correlation between the identified ceRNAs and SIC. The receiver operating characteristic (ROC) curve and area under the curve (AUC) values are used to compare diagnostic accuracy. The correlation between the relative expression levels of each gene and clinical parameters was assessed using either Pearson’s or Spearman’s correlation coefficient. *p* < 0.05 was considered statistically significant.

## 3 Results

### 3.1 Selection of SIC-related DEGs

**Figure 2:**
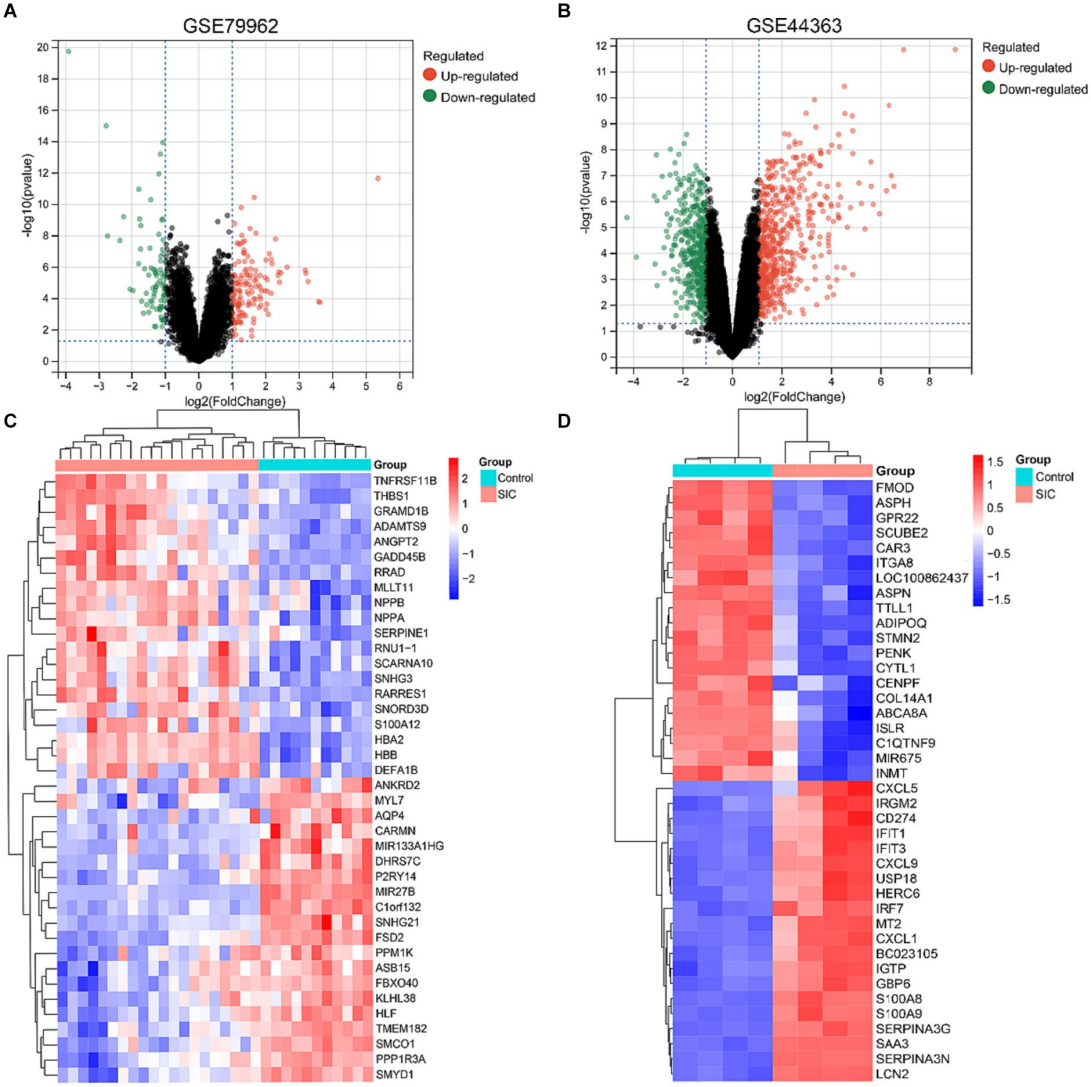
DEGs identified in GSE79962 and GSE44363. **(A)** Volcano plot of all DEGs in GSE79962. **(B)** Volcano plot of all DEGs in GSE44363. **(C)** Heatmap of the top 20 upregulated and downregulated DEGs in GSE79962. **(D)** Heatmap of the top 20 upregulated and downregulated DEGs in GSE44363.

Differential genes in datasets GSE79962 and GSE44363 were analyzed using GEO2R tool. All differential genes were visualized in volcano plots (Figures 2A,B), and the top 20 upregulated and downregulated genes were selected for generating a heatmap of differential genes (Figures 2C,D). A filter criterion with *p* < 0.05 and |logFC| > 1 was applied, resulting in the selection of 222 significantly DEGs in the GSE79962 dataset, which included 141 upregulated and 81 downregulated genes. In the GSE44363 dataset, a total of 930 significant DEGs were identified using the same criteria, with 539 genes upregulated and 391 genes downregulated. The intersection of 2 datasets was used to obtain 57 common DEGs (Supplementary Table 1: S2).

### 3.2 Functional enrichment analysis and PPI network construction

**Figure 3:**
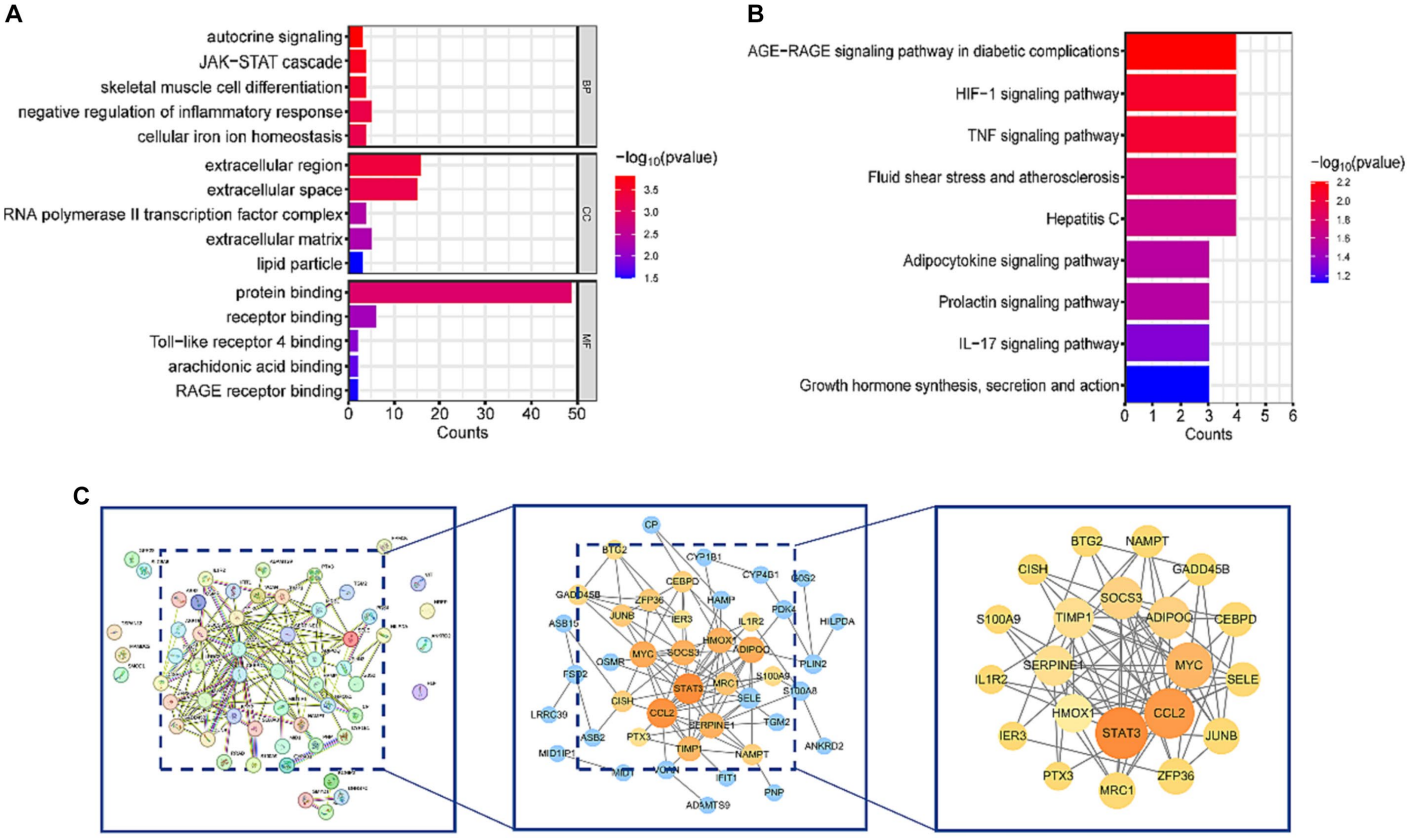
Functional enrichment analysis and PPI network of DEGs. **(A)** Results of GO analysis. **(B)** Results of KEGG analysis. **(C)** PPI network diagram of DEGs and hub genes. After removing isolated nodes, the orange and yellow nodes represent the top 50% of nodes ranked by the MCC algorithm. Nodes with larger diameters and darker colors indicate higher degrees in the PPI network.

DAVID database was used to perform KEGG and GO analyses on the 57 common DEGs. GO analysis revealed that DEGs were involved in several biological processes (BP), including autocrine signaling, JAK–STAT cascade, skeletal muscle cell differentiation, negative regulation of inflammatory response, and cellular iron ion homeostasis. The major molecular functions (MF) of DEGs included protein binding, receptor binding, Toll-like receptor 4 binding, arachidonic acid binding, and RAGE receptor binding. The cellular components (CC) mainly associated with DEGs were the extracellular region, extracellular space, and RNA polymerase II transcription factor complex (Figure 3A). KEGG enrichment analysis showed that DEGs were significantly enriched in pathways related to HIF-1, TNF, IL-17 signaling, AGE-RAGE signaling pathway in diabetic complications, and Fluid shear stress and atherosclerosis (Figure 3B). These pathways are closely associated with inflammatory responses. Detailed data are presented in Supplementary Table 1: S3, S4.

To gain a deeper understanding of the interactions among DEGs, we constructed a PPI network using STRING, resulting in a network with 57 nodes and 106 edges. Subsequently, this PPI network was imported into Cytoscape software, and isolated nodes were removed, resulting in a DEGs network with 43 nodes and 106 edges. o further identify the most specific DEGs within this network, we used the CytoHubba plugin for analysis and ranking. This plugin evaluates the importance of nodes in the PPI using 11 different node ranking methods (20). We selected the MCC method, which offers high sensitivity and specificity, and identified the top 50% of DEGs as hub genes (Figure 3C). The specific gene names are listed in Table 3.

**Table 3:** Hub genes ranked in the top 50% by CytoHubba.

Gene name	Description
STAT3	Signal transducer and activator of transcription 3
CCL2	C-C motif chemokine ligand 2
SERPINE1	Serpin family E member 1
TIMP1	TIMP metallopeptidase inhibitor 1
ADIPOQ	Adiponectin, C1Q and collagen domain containing
HMOX1	Heme oxygenase 1
SELE	Selectin E
MYC	V-myc avian myelocytomatosis viral oncogene homolog
SOCS3	Suppressor of cytokine signaling 3
NAMPT	Nicotinamide phosphoribosyltransferase
MRC1	Mannose receptor, C type 1
JUNB	JunB proto-oncogene, AP-1 transcription factor subunit
CEBPD	CCAAT/enhancer binding protein delta
ZFP36	ZFP36 ring finger protein
CISH	Cytokine inducible SH2 containing protein
GADD45B	Growth arrest and DNA damage inducible beta
BTG2	BTG anti-proliferation factor 2
IL1R2	Interleukin 1 receptor type 2
S100A9	S100 calcium binding protein A9
PTX3	Pentraxin 3
IER3	Immediate early response 3

### 3.3 Identification of glycolysis-related hub gene

**Figure 4:**
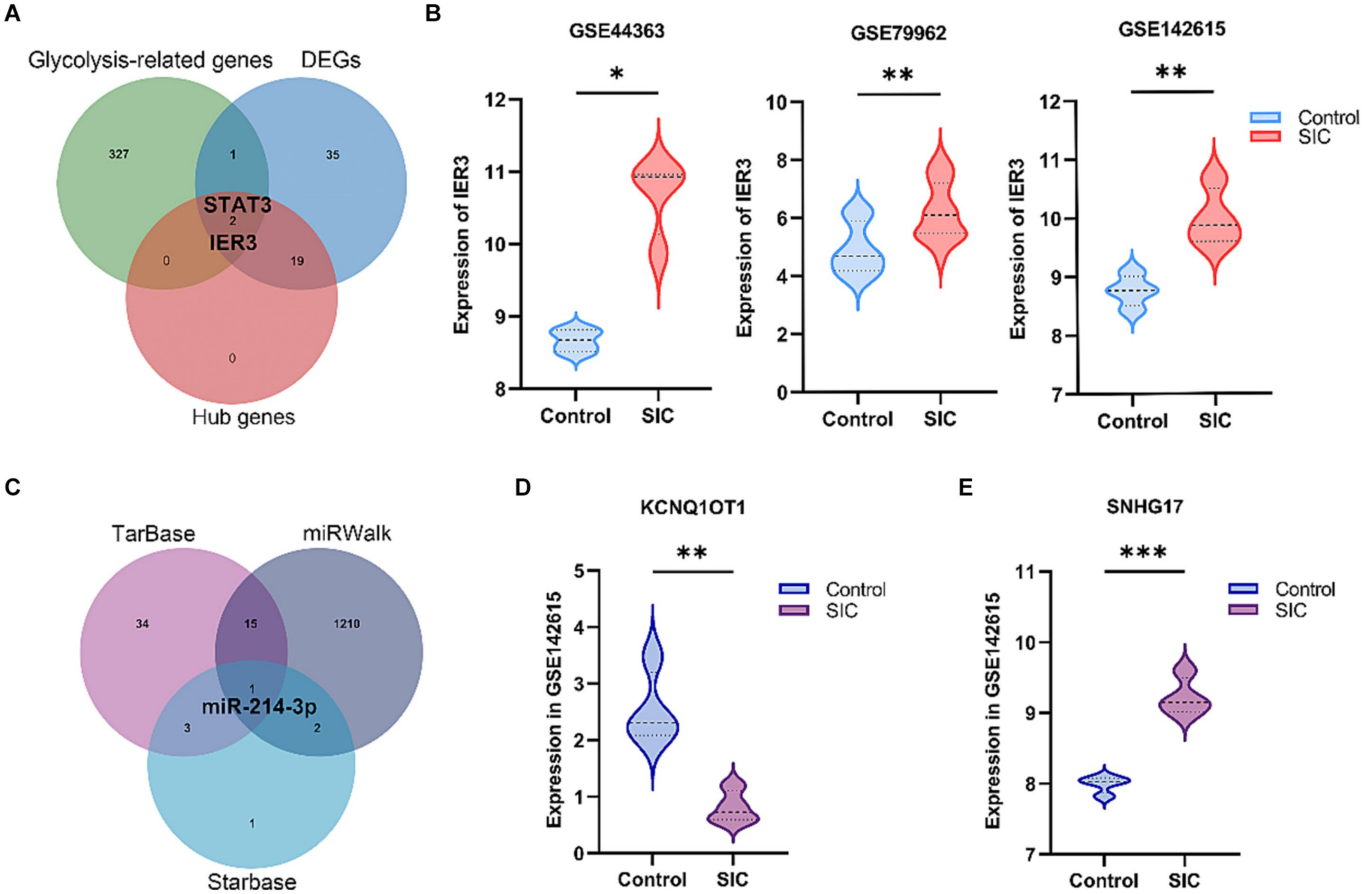
Selection of glycolysis-related DEGs and construction of ceRNA network. **(A)** Venn diagram results show the presence of 2 glycolysis-related hub genes (STAT3 and IER3). **(B)** Differential expression of IER3 in GSE79962, GSE44363, and GSE142615. **(C)** The common miRNA target identified in the 3 databases is miR-214-3p. **(D)** Expression of KCNQ1OT1 in GSE142615. **(E)** Expression of SNHG17 in GSE142615. *p < 0.05; **p < 0.01; ***p < 0.001.

A total of 330 glycolysis-related genes were obtained from the MSigDB database. Crossing these genes with the 57 DEGs and 21 hub genes led to the identification of two glycolysis-related DEGs, namely IER3 and STAT3 (Figure 4A). STAT3, as a well-known transcription factor, has been previously studied for its relevance to sepsis. IER3, a novel and specific gene discovered in this study, was selected as the glycolysis-related hub gene for further investigation. This gene showed significant upregulation in the SIC group in both GSE79962 and GSE44363 datasets. Validation was performed using the training dataset GSE142615, which confirmed that IER3 was also significantly upregulated in the SIC group of the GSE142615 dataset (Figure 4B).

### 3.4 ceRNA network construction

We predicted the miRNAs interacting with IER3 using the TarBase, starBase, and miRWalk databases (Supplementary Table 1: S5–S7). After intersecting the results from these three databases, we identified miR-214-3p (Figure 4C). Subsequently, we employed the RNA22 database to predict 3,748 lncRNAs that interact with miR-214-3p (Supplementary Table 1: S8). To validate the relevance of these lncRNAs to SIC, we utilized the GSE142615 dataset, which includes 670 differentially expressed lncRNAs. By taking the intersection, we identified 2 lncRNAs for validation: SNHG17 and KCNQ1OT1. Among them, SNHG17 was upregulated in the SIC group (logFC = 1.22764595) (Figure 4D), while KCNQ1OT1 was downregulated in the SIC group (logFC = −1.72168747) (Figure 4E). According to the theory of ceRNA, when the expression of lncRNAs is upregulated in SIC, they can better serve as “sponges” that competitively bind with miRNAs. Therefore, we chose SNHG17 and constructed a ceRNA network related to glycolysis: SNHG17/miR-214-3p/IER3.

### 3.5 Validation of gene-specific binding through dual-luciferase activity

**Figure 5:**
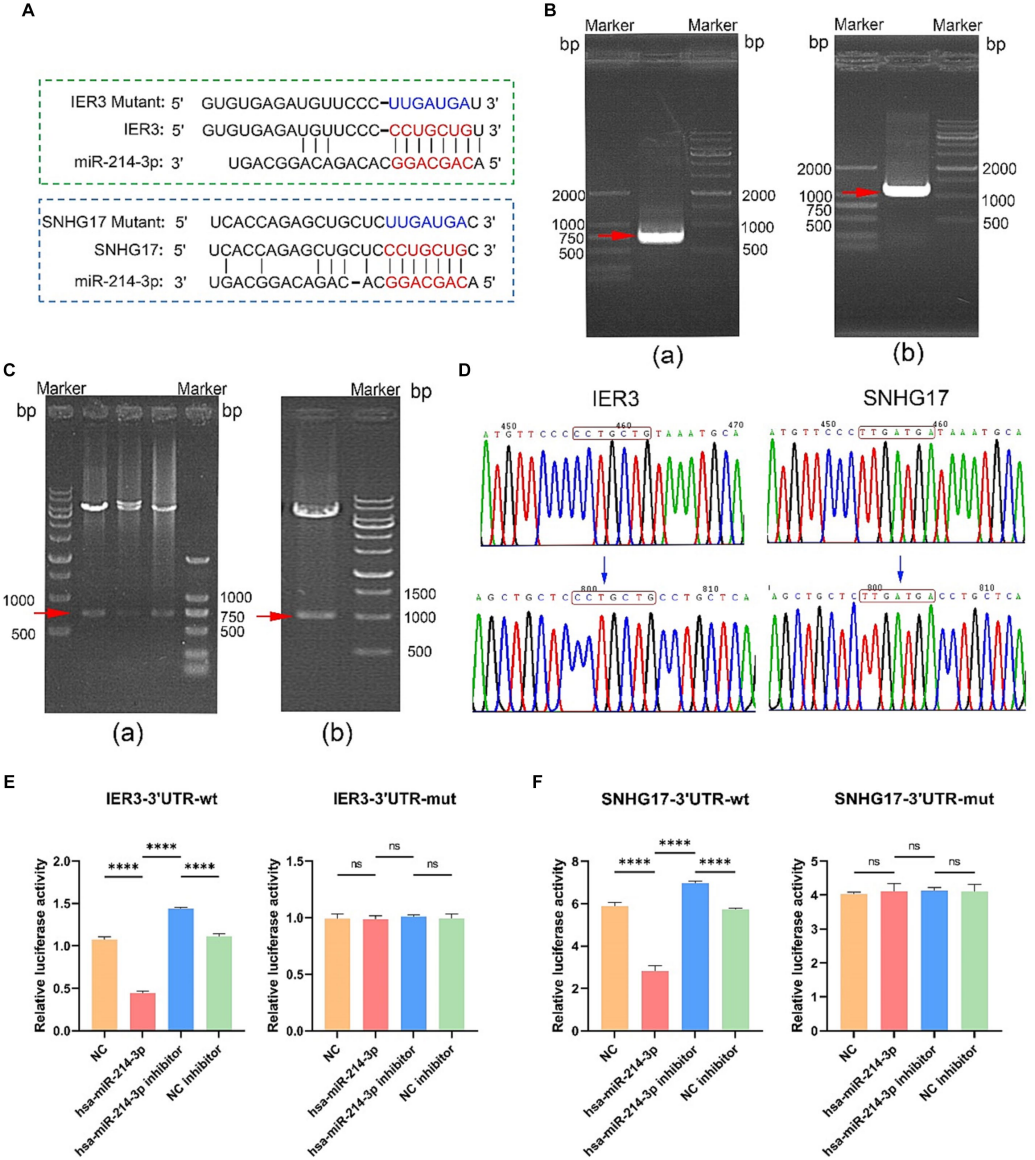
Dual-luciferase reporter gene assay. **(A)** Binding sites of miR-214-3p with IER3 and SNHG17, along with the sequences after mutation. **(B)** Amplification confirmation of PCR products. (a) IER3-3'UTR-wt PCR identification results (737bp); (b) SNHG17-3'UTR-wt PCR identification results (1086 bp). **(C)** Identification of plasmid enzyme digestion products. (a) IER3 plasmid enzyme digestion products (737bp); (b) Identification results of SNHG17 plasmid enzyme digestion products (1086bp). **(D)** Sequencing results of wild-type and mutant plasmids. The wild-type plasmids of IER3 and SNHG17 are consistent with the reference sequence, while the corresponding positions of mutant plasmids have been successfully mutated. **(E,F)** Dual-luciferase reporter gene assay results. ****p < 0.0001, ns: *p* > 0.05.

Potential binding sites between IER3 and miR-214-3p and miR-214-3p and SNHG17 were predicted using the TargetScan website. PCR primers were designed based on the 3’UTR sequences of IER3 and SNHG17, and both wild-type and mutant gene sequences were synthesized (Figure 5A). Successful amplification of the PCR products for IER3 and SNHG17 was confirmed by agarose gel electrophoresis (Figure 5B). After PCR product recovery, enzymatic digestion, ligation, and transformation, plasmids were extracted and identified as positive clones by enzymatic digestion (Figure 5C). Subsequent plasmid sequencing results revealed that the wild-type plasmids (IER3-3′UTR-wt, SNHG17-3′UTR-wt) had sequences identical to the reference sequence. In contrast, mutant plasmids (IER3-3′UTR-mut and SNHG17-3′UTR-mut) exhibited a mutation from CCTGCTG to TTGATGA in both cases (Figure 5D).

In the wild-type IER3 group, relative luciferase activity significantly decreased in the IER3-3′UTR-wt + miR-214-3p group compared to the empty plasmid NC group. Conversely, the IER3-3′UTR-wt + miR-214-3p inhibitor group showed a significant increase in relative luciferase activity compared to the NC inhibitor group. No significant change in relative luciferase activity was observed in the IER3-3′UTR-mut group, indicating that miR-214-3p can specifically bind to the 3′UTR target site of the IER3 gene, and there is only one binding site (Figure 5E). In the wild-type SNHG17 group, relative luciferase activity significantly decreased in the SNHG17-3′UTR-wt + miR-214-3p group compared to the empty plasmid NC group. Conversely, the SNHG17-3′UTR-wt + miR-214-3p inhibitor group showed a significant increase in relative luciferase activity compared to the NC inhibitor group. No significant change in relative luciferase activity was observed in the SNHG17-3′UTR-mut group, indicating that miR-214-3p can specifically bind to the 3′UTR target site of the SNHG17 gene, and there is only one binding site (Figure 5F). All the raw data is presented in Supplementary Table S2.

### 3.6 Validation of the ceRNA network by PBMC samples

**Figure 6:**
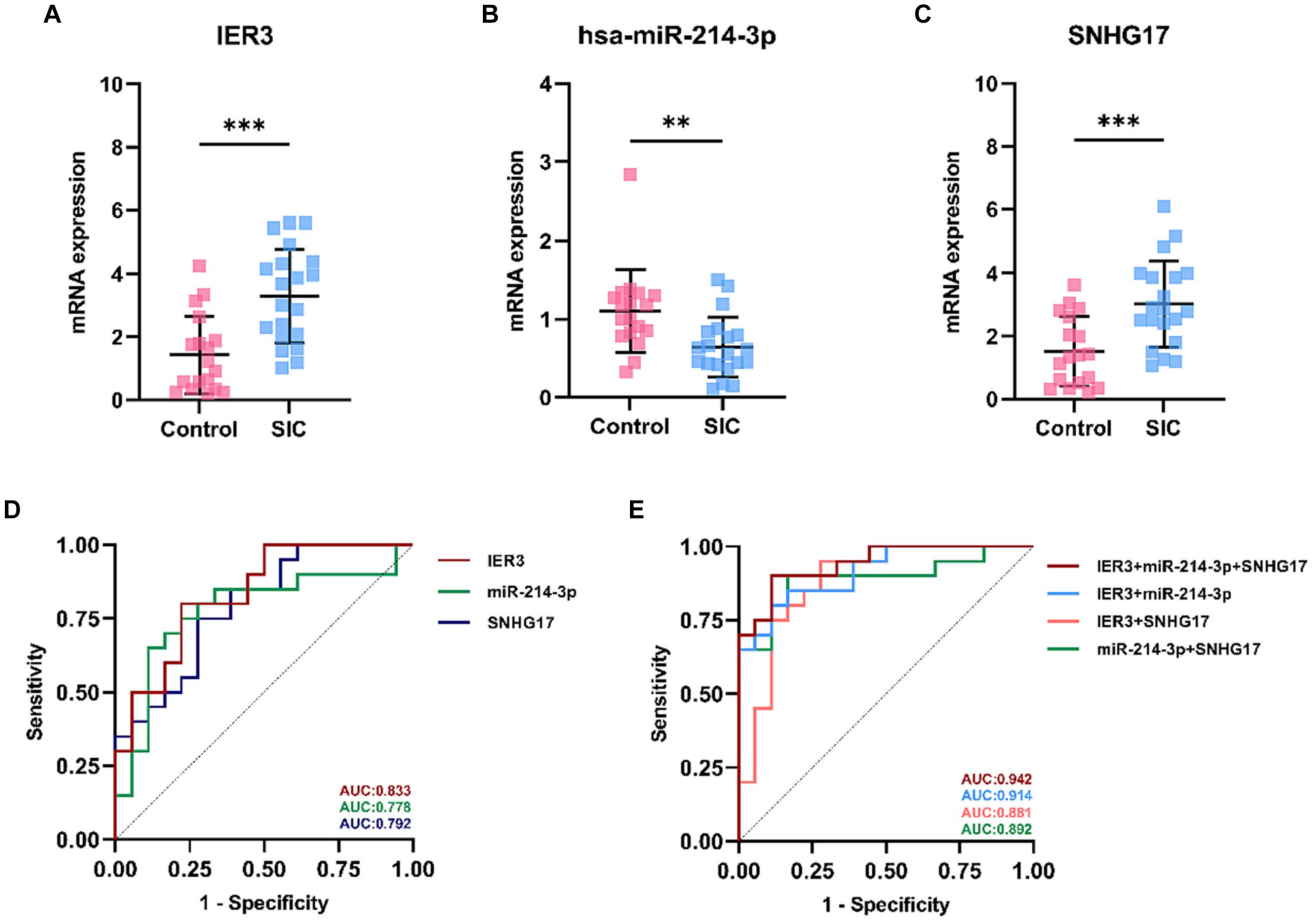
Expression and ROC curve of various genes in SIC patients. **(A)** Relative expression levels of IER3 in PBMCs of SIC patients. **(B)** Relative expression levels of miR-214-3p in PBMCs of SIC patients. **(C)** Relative expression levels of SNHG17 in PBMCs of SIC patients. **(D)** ROC curves assessing the individual diagnostic ability of IER3, miR-214-3p, and SNHG17 for SIC. **(E)** ROC curves assessing the combined diagnostic ability of IER3, miR-214-3p, and SNHG17 for SIC.**p < 0.01,***p < 0.001.

In this study, a total of 20 patients meeting the criteria for SIC were included, and an additional 18 healthy individuals were recruited as the control group. Detailed clinical data for SIC patients can be found in Supplementary Table 3: S2. The expression of SNHG17/miR-214-3p/IER3 in PBMCs of SIC patients was detected using RT-qPCR (Supplementary Table 3: S1). The results showed that the expression of IER3 and SNHG17 was significantly upregulated in PBMC samples from SIC patients (*p* < 0.05) (Figures 6A,C), while miR-214-3p was downregulated (*p* < 0.05) (Figure 6B). Logistic regression analysis showed a significant correlation between IER3, miR-214-3p, SNHG17 expression and SIC (Table 4). ROC curve analysis showed that IER3 (AUC: 0.833), miR-214-3p (AUC: 0.778), and SNHG17 (AUC: 0.792) had good diagnostic capabilities (Figure 6D). Subsequently, we combined various indicators and predictive ability of the model again, the multivariate ROC analysis revealed that the combined model of IER3 + miR-214-3p + SNHG17 had the best diagnostic performance (AUC: 0.942), followed by the models combining two genes: IER3 + miR-214 (AUC: 0.914), IER3 + SNHG17 (AUC: 0.881), and miR-214-3p + SNHG17 (AUC: 0.892), all of which exhibited higher diagnostic capabilities than the single-gene models (Figure 6E). We used the Spearman correlation coefficient to examine the correlation between the relative expression levels of each gene and clinical indicators in patients. It was found that the relative expression level of IER3 was negatively correlated with the oxygenation index (*p* < 0.05), and the relative expression level of miR-214-3p was negatively correlated with NT-proBNP (N-terminal pro-brain natriuretic peptide) (*p* < 0.05) (Table 5).

**Table 4:** Univariate and multivariate logistic analyses in SIC patients.

Variables	Univariate analysis			Multivariate analysis		
	OR	95% CI	*p* value	OR	95% CI	*p* value
IER3	2.352	1.314–4.208	0.004	2.329	1.042–5.203	0.039
miR-214-3p	0.062	0.08–0.497	0.009	0.02	0.001–0.423	0.012
SNHG17	2.738	1.367–5.483	0.009	2.8	1.055–7.428	0.039

CI, confidence interval; OR, odds ratio.

**Table 5:** Correlation of IER3, miR-214-3p, and SNHG17 with clinical parameters.

Clinical parameters	IER3		miR-214-3p		SNHG17	
	Correlation coefficient	*p* value	Correlation coefficient	*p* value	Correlation coefficient	*p* value
Age	−0.044	0.853	0.269	0.251	−0.214	0.366
NT-proBNP	0.044	0.854	−0.457*	**0.043**	0.3	0.199
Oxygenation- Index	−0.468*	**0.037**	−0.036	0.879	0.115	0.629
LVEF	0.297	0.204	0.242	0.904	0.014	0.954
PCT	−0.259	0.269	−0.125	0.6	0.238	0.311
WBC	0.28	0.232	0.128	0.591	0.033	0.89
NEU	0.107	0.654	−0.137	0.565	0.104	0.663
Platelets	0.119	0.617	−0.165	0.487	0.164	0.489
Creatinine	0.02	0.932	−0.132	0.578	−0.076	0.75
Total bilirubin	0.156	0.512	−0.128	0.591	0.354	0.126

NT-proBNP, N-terminal pro-brain natriuretic peptide; LVEF, Left ventricular ejection fraction; PCT, Procalcitonin; WBC, White blood cell count; NEU, Neutrophil count. *p < 0.05. Bold font indicates that this correlation coefficient *p* value is statistically different.

## 4 Discussion

Sepsis is a disease characterized by a dysregulated host response to infection, resulting in multi-organ dysfunction (1), and it claims the lives of millions of people worldwide each year (29). SIC is a severe complication resulting from sepsis, often indicating a poorer prognosis and a higher mortality rate. Due to the urgency of diagnosing and treating SIC, there is a clinical need for highly specific diagnostic tools to promptly recognize the condition.

Currently, echocardiography and biomarkers of myocardial injury are the preferred modalities for clinical assessment, but they are not specific enough to diagnose SIC. A significant issue is that reduced afterload resulting from the distributive shock may pseudo-normalize a depressed EF (coupling between contractility and afterload) (30, 31). While echocardiographic parameters like diastolic function and right ventricular (RV) systolic function lack the same specificity as LVEF in diagnosing SIC, they often require exclusion in conjunction with other diagnostic methods (6). Novel parameters such as global longitudinal strain (GLS), myocardial performance index (MPI) currently lack reliability data in clinical applications (32). Therefore, exploring the pathophysiological mechanisms of SIC and identifying indicators that are more sensitive and specific will provide robust assistance in the diagnosis and treatment of SIC.

The pathogenesis of septic cardiomyopathy is complex, with metabolic changes playing a pivotal role (33). During sepsis, there is a shift in cellular metabolism from oxidative phosphorylation to glycolysis, a phenomenon known as the Warburg effect (34). Enhanced glycolysis can lead to rapid activation of immune cells, resulting in the release of numerous pro-inflammatory cytokines. In some cases, this process can trigger a “cytokine storm,” further exacerbating organ dysfunction (4). Studies have shown that inhibiting glycolysis with 2-deoxyglucose (2-DG) significantly alleviates cardiac dysfunction and improves survival rates in septic mice. Additionally, this intervention enhances the expression of Sirt1 and Sirt3, which are associated with mitochondrial function protection in cardiac muscle, while suppressing the expression of apoptotic genes Bak and Bax, as well as JNK phosphorylation (12). These findings underscore the close relationship between glycolysis and SIC. However, the specific mechanisms by which glycolysis operates in the context of SIC remain to be fully elucidated, warranting further in-depth research.

With the development of genomics technology in recent years, sepsis diagnosis and treatment have benefited from the use of both gene sequencing and gene therapy (35, 36). The discovery of novel biomarkers through genomic sequencing techniques has provided new avenues for identifying diagnostic targets in diseases. Analyzing differential gene expression from datasets such as GEO and constructing ceRNA networks has emerged as a crucial approach in current research.

In this study, we used bioinformatics techniques to identify 2 hub genes related to glycolysis in SIC: IER3 and STAT3. STAT3, as a classical transcription factor, plays a crucial role in regulating various physiological pathways, including cell growth, differentiation, and apoptosis. Previous research has confirmed the pivotal role of STAT3 in LPS-induced myocardial dysfunction (37). IER3, also known as IEX-1, is a stress-inducible immediate-early gene It plays a role in influencing mitochondrial F1Fo ATPase activity, regulating mitochondrial reactive oxygen species balance, and participating in the modulation of mitochondrial oxidative phosphorylation and glycolysis (38). IER3 has a unique role in the pathogenesis of cardiovascular and inflammatory diseases. Its expression is significantly upregulated in the myocardial tissues of mice subjected to pressure overload, and IER3 gene knockout may lead to hypertension and cardiac hypertrophy in mice (39). It can impact inflammatory responses by regulating pathways such as NF-κB and Nrf2 (40). In this study, we revealed a relationship between IER3 and SIC for the first time and gained new insights into the study of IER3.

Subsequently, We then predicted miRNAs targeting IER3 from multiple databases and identified miR-214-3p as one of the miRNAs targeting IER3. Previous research has suggested a potential link between miR-214-3p and the pathogenesis of SIC. Overexpression of miR-214-3p in septic mice models has been shown to alleviate myocardial dysfunction and damage. Additionally, it inhibits myocardial inflammation, and reduce autophagy (41). Upregulation of miR-214-3p has an inhibitory effect on myocardial cell apoptosis and injury in rats with myocardial ischemia/reperfusion injury (42). Conversely, its deficiency may exacerbate cardiac fibrosis (43). We then predicted lncRNAs targeted by miR-214-3p, among which SNHG17 was identified. Studies have indicated that SNHG17 is upregulated in ovarian cancer and acts as a molecular sponge for miR-214-3p, relieving miR-214-3p’s inhibitory effect on the cell cycle regulator CDK6, thereby promoting the growth of ovarian cancer cells (44). SNHG17 is upregulated in various tumors and is closely associated with adverse prognosis and advanced clinical-pathological characteristics in cancer patients (45). However, its role in cardiovascular diseases has not been thoroughly investigated.

Based on the above research, we predicted and established a novel ceRNA network, SNHG17/miR-214-3p/IER3. To validate the authenticity of this network, we conducted a luciferase assay, confirming the specific binding relationships between IER3/miR-214-3p and SNHG17/miR-214-3p. The unique expression of IER3, miR-214-3p, and SNHG17 was validated by qPCR utilizing PBMC samples from clinical SIC patients. The ROC curves demonstrate that this ceRNA network possesses a strong diagnostic capability. Interestingly, we also observed correlations between IER3 and oxygenation index, as well as miR-214-3p and NT-proBNP.

It should be noted that in this study, a direct correlation between IER3-miR-214-3p-SNHG17 and LVEF was not observed. This finding is similar to previous research by Parker et al. (46), who reported that only about 50% of patients with septic shock had a reduced LVEF. Additionally, survivors had a lower LVEF on average compared to non-survivors (46). While a decreased LVEF is a clinical diagnostic criterion for SIC, it is important to note that LVEF values can be influenced by cardiac loading conditions and vary with individual differences in filling pressures and cardiac afterload. Therefore, the specificity of LVEF values in diagnosing and prognosticating SIC is suboptimal. Although IER3-miR-214-3p-SNHG17 did not show a direct correlation with LVEF, its expression in SIC and the sensitivity of this diagnostic model still suggest the potential diagnostic value of this network. These findings are significant for understanding SIC’s pathophysiology and identifying possible treatment options.

In this study, we have established a novel glycolysis-related ceRNA network, SNHG17/miR-214-3p/IER3, which has not been previously reported in current studies, and discovered the precise expression of IER3 in SIC for the first time. To increase the credibility of our results, we experimentally validated the specific binding of this ceRNA network through dual-luciferase reporter assays and confirmed the differential expression of SNHG17/miR-214-3p/IER3 in PBMCs using external datasets and human PBMC samples. Nevertheless, this study has several limitations. Firstly, the sample sizes from the selected datasets were restricted due to the scarcity of SIC samples in the GEO database. Additionally, the number of participants included in our PBMC validation was relatively small, requiring further clinical research to validate and broaden the applicability of our findings. Furthermore, considering the restricted specificity of LVEF in SIC diagnosis and prognosis, incorporating additional novel biomarkers like global longitudinal strain (GLS) and myocardial performance index (MPI) during the collection of clinical cases could be beneficial. Alternatively, applying stricter inclusion criteria, such as LVEF < 45% (47–49), for the combined diagnosis of SIC might enhance the accuracy and specificity of SIC diagnosis. In future studies, we will employ more rigorous diagnostic criteria for the collection of clinical cases and further validate the accuracy of this ceRNA network in diagnosing and prognosticating SIC.

## 6 Conclusion

We have identified IER3 as a novel target related to glycolysis in SIC and established a new ceRNA network: SNHG17/miR-214-3p/IER3. This ceRNA network may be closely associated with the development and occurrence of SIC. Despite certain limitations, this study opens up new avenues for a more profound understanding of the pathophysiological mechanisms of SIC and the development of more effective diagnostic tools. Future research will require more rigorous and extensive clinical studies to validate its diagnostic potential in clinical settings.

## Data availability statement

The original contributions presented in the study are included in the article/Supplementary material, further inquiries can be directed to the corresponding authors.

## Ethics statement

The studies involving humans were approved by The Ethics Committee of Panyu Central Hospital, Guangzhou, China. The studies were conducted in accordance with the local legislation and institutional requirements. The participants provided their written informed consent to participate in this study. Written informed consent was obtained from the individual(s) for the publication of any potentially identifiable images or data included in this article.

## Author contributions

LC: Conceptualization, Software, Validation, Visualization, Writing – original draft. JL: Validation, Writing – original draft. FX: Methodology, Validation, Writing – original draft. ZH: Methodology, Writing – original draft. WL: Methodology, Writing – original draft. HC: Funding acquisition, Project administration, Resources, Writing – review & editing. JH: Funding acquisition, Project administration, Resources, Writing – review & editing.

## Footnotes

^1^ https://www.ncbi.nlm.nih.gov/geo/

^2^ http://www.ncbi.nlm.nih.gov/geo/geo2r/

^3^ https://jvenn.toulouse.inrae.fr/app/example.html

^4^ https://david.ncifcrf.gov/tools.jsp

^5^ https://string-db.org

^6^ https://cytoscape.org/

^7^ https://www.gsea-msigdb.org/gsea/msigdb/

^8^ https://dianalab.e-ce.uth.gr/html/diana/web/index.php?r=tarbasev8

^9^ http://starbase.sysu.edu.cn/

^10^ http://mirwalk.umm.uni-heidelberg.de/

^11^ https://cm.jefferson.edu/rna22/Precomputed/

^12^ http://www.Targetscan.org
